# Marketing management practices in an emergency care department

**DOI:** 10.25122/jml-2021-0059

**Published:** 2021

**Authors:** Ligia Sanda Marinela Pop-Micle, Norbert Dacian Stenczel, Victor Lorin Purcarea, Iuliana-Raluca Gheorghe, Gabriela Hoffer Oniga

**Affiliations:** 1. Department of Healthcare Marketing and Medical Technology, Carol Davila University of Medicine and Pharmacy, Bucurest, Romania

**Keywords:** marketing management, emergency room (ER), physicians, marketing

## Abstract

The aim of this case study was to identify effective marketing management strategies in the Emergency Care Department of a Romanian Emergency Hospital. An observational design study was conducted, and the instrument for collecting the data was the self-administered questionnaire. Out of 100 questionnaires completed, 74 proved to be valid. The statistical analysis was performed using the IBM SPSS Statistics software version 20 and Microsoft Office Excel 2013. Quantitative variables were described by means and standard deviations, whereas for the qualitative variables, frequencies and percentages were used. Most of the respondents were aged between 28 and 35 years (33.8%) and women (60.8%). The emergency room (ER) physicians identified the following factors as being important in becoming very good doctors: continuous specialization (81.1%), reading medical literature (78.4%), and getting involved in more complicated cases (78.4%). The ER physicians mentioned that most of their patients were satisfied with the medical information received (31.1%), properly understood the medical information received (18.9%), and 68.9% of the doctors considered that patients were showing them total respect. Although 40.5% of the ER physicians declared they were suffering from burnout, 25.7% felt satisfied and joyful in their daily activities. Today’s context triggered the need to integrate the concept of consumer value-driven care in the health care system, especially in the ER departments, by implementing the principles of efficient marketing management practices.

## Introduction

Marketing focuses on the connection between two central elements: the product and the market since the main goal of the marketer is to establish a link between them. The first challenge for health marketers is to design the “product” that, in this case, should respond to keywords such as “health”, “effective results”, and “quality of life”. The second challenge is to settle to a target population, especially in healthcare, where services are sold to a wide range of people. As the industry has matured, health managers have realized that they cannot apply mass production, and the market particularities should be identified (the greater the interest in that service, the more the market turns into a niche). In any case, marketers must have the ability to correctly segment the audience that requires a healthcare service directly or indirectly.

Before an organization can implement an effective marketing plan, it must first develop its strategy through basic planning, such as creating a vision, setting a mission, identifying key resources, and setting goals [1]. Through this activity process, the organization identifies stakeholders and consumers to ensure proper development and implementation.

Developing consumer awareness, improving the credibility and the image of the hospital, raising the reputation, serving as an information resource, improving market activity, influencing consumer decision-making are just some reasons to apply the principles of marketing in healthcare services [2].

When medical organizations offer services that are not useful or needed, they reflect other services, such as hospital on-call rooms, that are legally mandated and very popular as they accept any patient [2].

Due to the unplanned nature of the patient’s presence, an emergency medical unit must provide initial treatment for a wide range of diseases and injuries, some of which can be life-threatening and require immediate attention. In some countries, emergency departments have become important entry points for those without other means of access to health care. Hospital emergency rooms (ER) operate 24/7, although the number of employees may vary to the increased presence of patients [3].

ER requires different equipment and different approaches than most other hospital departments. Often, patients end up in unstable conditions and need to be treated quickly. Moreover, they may be unconscious, and physicians may not have essential information about them, such as medical history, allergies, and blood group. However, the ER staff is trained to work quickly and efficiently, even with minimal information. Hence, ER efficiency measurement scales can be grouped into three main categories: volume, time, and patient satisfaction [4, 5]. Volume refers to the hourly arrivals, the percentage of beds occupied in the ER, and the age of patients who require urgent medical attendance. Time is reflected in the activities of evaluating and monitoring the efficiency of the process, and these are less widespread, as an active effort is needed to collect and analyze data, especially in the ER. Patient satisfaction is useful in measuring the trend of the already collected perceived information about the physicians and the overall hospital-provided services, mainly in terms of changes in patient perceptions over time [6]. Because patient satisfaction measures are very different and subjective in nature, they are less helpful in improving primary processes, such as ER care. Exchanges of health information can reduce unpleasant visits to ER by providing current data on hospitalization, discharge, or transfers to other healthcare facilities, thus enabling people to use ER as a primary care form.

The objective of marketing within the ER is to simultaneously assess both the objectives of the organization and of the beneficiary, meaning the patients. If the objectives of both parties are not met, the marketing process will be considered unsuccessful. The challenge for marketers is to identify the objectives of the two parties and facilitate their achievement in a mutually beneficial way, including the establishment and enhancing the organization’s reputation [7].

Working in an emergency unit requires rigorous assessment skills, flexibility, and the ability to operate in a high-stress environment. As such, the main goal of the ER staff is to stabilize the patient’s condition by treating the acute problem. Thus, this research aimed to identify effective marketing management strategies in the Emergency Care Department of a Romanian Emergency Hospital.

The specific objectives of this case study were:

• To identify the demographic and professional profile of physicians in the ER (age, gender);
• To determine the way physicians define a “good” and “very good” ER doctor, what would be the influencing factors of these statuses, as well as their opinions on the organizational factors in becoming a good ER physician;
• To highlight the distribution of physicians’ perceptions of their involvement in the patient care, the perceived level of patient satisfaction from their perspectives, and the possible reasons for patients to return for a follow-up;
• To assess the level of the required training of ER physicians connected to their perceived burnout levels;
• To ascertain the importance given by ER physicians to their professional training in relation to their age.

## Material and Methods

An observational design study was conducted by collecting data through self-administered questionnaires.

The questionnaires consisted of 5 questions referring to both demographic data and professional training data, such as age, gender, medical specialty, professional degree, and 16 specific marketing management of ER services that focused on patient satisfaction and their emotions, but also on the perceived level of the specialized staff. Each question was measured on a 5-point Likert scale, ranging from “totally disagree – 1” to “totally agree – 5”.

Consequently, out of 100 participants, 74 questionnaires proved to be valid. The inclusion criteria were the following: ER physicians were employed within an ER of an Emergency Hospital in Romania, had continuous professional activity, and had no specific mental illnesses.

The statistical analysis was performed using the IBM SPSS Statistics software version 20 and Microsoft Office Excel 2013. Quantitative variables were tested for distribution using the Shapiro-Wilk test and were expressed as means with standard deviations, and the categorical variables were illustrated as percentages or frequencies.

For objective 5, the independent quantitative variables were tested using the Kruskal-Wallis H test as their distribution was non-parametric. The Dunn-Bonferroni tests were post-hoc tests that were performed to have a more in-depth overview of the results.

## Results

### Results of objective 1

The data in Table 1 and Table 2 represent the distribution of physicians by age and gender. It can be observed that most of the participants were young physicians, with ages between 28 and 35 years (33.8%) and 36–44 years (24.3%), and women (60.8%).

**Table 1.** The distribution of physicians by age.

**Age category**	**No.**	**Percentage**
**28–35 years**	25	33.8%
**36–44 years**	18	24.3%
**45–53 years**	16	21.6%
**54–65 years**	15	20.3%

**Table 2.** The distribution of physicians by gender.

Gender	No.	Percentage
**Female**	45	60.8%
**Male**	29	39.2%

Table 3 shows the distribution of physicians by their professional degree. There was an equal distribution of physicians by their professional degree but with a predominance of primary care physicians (56.8%).

**Table 3.** The distribution of physicians by their professional degree.

Professional Degree	No.	Percentage
**Specialist physician**	32	43.2%
**Primary physician**	42	56.8%

### Results of objective 2

The information collected about the ER physicians’ perceptions regarding the status of being a very good physician revealed that 81.1% of the doctors considered that continuous specialization is a very important factor, 78.4% considered that constantly reading medical literature is a very important factor to be a good doctor, 78.4% of the doctors believed that involvement in more complicated cases is an essential factor, and 81.1% of the doctors considered that discussing with physicians from other specialties is also a very important factor.

Moreover, 47.3% of the ER physicians stated that they totally agreed that the Emergency Hospital offered all the necessary support to become a good physician.

### Results of objective 3

The distribution of the ER physicians in relation to the degree of importance offered to the patient emphasized that 79.7% of them considered they offered enough time and importance to the anamnesis and the clinical examination of the patients, 90.5% of doctors considered that they offered enough time and importance to inform the patient about the diagnosis and treatment, and 85.1% of the doctors considered that they offered enough time and importance to inform the patients’ relatives regarding their conditions and improvements.

The distribution of the ER physicians in relation to the degree of agreement related to the achieved patient satisfaction highlighted that 31.1% of them fully considered that patients were satisfied with the medical care received. Only 18.9% noticed that patients properly understood the medical information received, and 68.9% of the doctors considered that patients were fully respected by their colleagues. Also, only 20.3% considered that their patients did not show compliance to the indicated treatment.

Furthermore, the distribution of the ER physicians in relation to their level of involvement revealed that 74.3% of them totally considered that they used simple phrases and words adapted to the patients’ level of understanding, 79.7% explained clearly the patients’ treatment and diagnosis, and 87.8% of the doctors informed the patients that they have the right to a second opinion. Only 31.1% of the doctors considered that their patients return for follow-up.

The distribution of the ER physicians in relation to the patients’ intentions to return for a follow-up or readmission in the healthcare organization emphasized that 59.5% of them totally agreed that the way doctors address the patient and relatives is an essential factor in achieving compliance and 35.1% considered that the level of knowledge compared to other colleagues is an important factor for the patients’ willingness for follow-up. Moreover, 54.1% of the ER physicians totally considered that the recommendation of former patients or family doctors is an important factor, 64.9% considered that the professionalism and interest shown are important factors, and only 23% of the doctors considered that emotional detachment is an important variable for the patients’ follow-up.

### Results of objective 4

The distribution of ER physicians in relation to the level of co-workers’ training revealed that 55.4% of them believed that a very good level of training is similar to the one found in big cities, and 54.1% agreed that their colleagues inspire confidence and professionalism in patients and other doctors. In comparison, 52.7% of the ER doctors stated that most colleagues are aware of new treatment methods, 50% strongly believed that most colleagues might cope with a difficult case, and 52.7% highlighted that most of their colleagues might realize when advanced medical care is needed.

**Figure 1.**
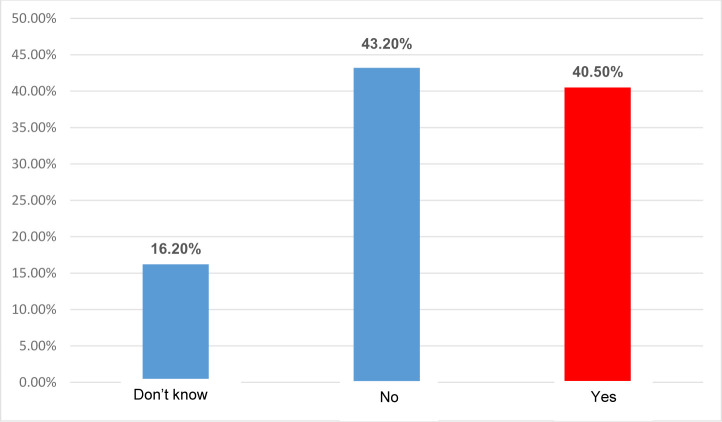
Distribution of the ER physicians who suffered from burnout (emotional exhaustion).

Figure 1 illustrates the distribution of the ER physicians who believed they suffered from burnout (emotional exhaustion). As such, 40.5% of the physicians declared they suffered from emotional exhaustion.

The data in Table 4 describe the emotions of the ER physicians perceived in the healthcare organizations. Thus, 28.4% of the ER doctors felt stressed and tired in the healthcare organization, but, at the same time, respected by the patients, and 25.7% of the doctors felt satisfied and joyful in their daily activities.

**Table 4.** The distribution of the ER physicians’ perceived emotions in the healthcare organization they work for.

Answer	No./Percentage				
	No	Rather no	Neither yes, nor no	Rather yes	Yes
**I feel stressed and tired**	6 (8.1%)	8 (10.8%)	17 (23%)	22 (29.7%)	21 (28.4%)
**I feel respected by the patients**	1 (1.4%)	11 (14.9%)	14 (18.9%)	27 (36.5%)	21 (28.4%)
**I feel satisfied and joyful**	2 (2.7%)	12 (16.2%)	20 (27%)	21 (28.4%)	19 (25.7%)

### Results of objective 5

The data in Table 5 show the comparison of the professional training scores in relation to the age of the ER physicians. The professional training scores required to be a very good doctor were computed according to the following scale: for total agreement – 5 points, partially agreement – 4 points, neither agreement nor disagreement – 3 points, partially disagreement – 2 points, total disagreement – 1 point. The scores had non-parametric distributions, according to the Shapiro-Wilk test (p<0.05), so the reporting of the results used the registered average scores. The Kruskal-Wallis H test demonstrated statistically significant differences in scores between the ER physicians by age (p=0.007). The post-hoc testing showed that physicians aged 54–65 gave more significant importance to the level of training (average rank = 46.9) compared to the doctors aged 36–44 years (average rank = 27.03) (p=0.009).

**Table 5.** Post-hoc comparison of the professional training scores in relation to the age of the ER physicians.

Age category*	28–35 years	36–44 years	45–53 years	54–65 years
**28–35 years**	-	0.662	1.000	0.348
**36–44 years**	0.662	-	0.054	0.009
**45–53 years**	1.000	0.054	-	1.000
**54–65 years**	0.348	0.009	1.000	-

* Dunn-Bonferroni Post-Hoc Test.

## Discussion

The results of objective 1 revealed that most of the participating ER physicians were young, with ages between 28 and 35 years (33.8%) and 36–44 years (24.3%). Also, 60.8% were females, and primary care physicians were predominant (56.8%).

Regarding objective 2, the distribution of the ER physicians in relation to what is necessary to be a very good doctor, 81.1% of them considered that continuous specialization is a significant factor, followed by 78.4% of the physicians who considered that constantly reading medical literature is also a critical factor in becoming a good doctor, while 78.4% of the doctors considered that involvement in more complicated cases is an important factor and 81.1% of the doctors considered that discussing with their colleagues from other specialties is essential to become a good doctor. Moreover, 47.3% of the ER physicians stated that they think that the healthcare organizations they work for offer the necessary support to become a very good doctor.

The opinions of the ER physicians regarding the achieved satisfaction of the patients from a doctor’s perspective when coming to the ER were the following: 31.1% of the doctors fully considered that patients were satisfied with the medical care received, and only 18.9% noticed that patients understood the provided medical information, whereas 68.9% of the doctors considered that patients were fully respected by their specialized colleagues and only 20.3% agreed that their patients did not show any compliance to the recommended treatment.

The results of objective 3 revealed that 74.3% of the ER physicians totally agreed with the fact that they used sentences and words appropriate to the patient’s understanding, and that 79.7% of the doctors clearly explained the patient’s treatment and diagnosis, 87.8% of the physicians informed the patient that they were entitled to a second opinion and only 31.1% of the doctors considered that their patients come back for treatment scheme explanation. Moreover, 55.4% of the doctors stated that their training and their colleagues’ were similar to those found in big cities, and 54.1% considered that their colleagues inspired confidence and professionalism in both patients and doctors. Only 40.5% of the doctors considered that they had burnout (emotional exhaustion).

In order to avoid getting the ER of an Emergency Hospital crowded and overused, some management strategies should be implemented. Some examples of the ideal ER services usage are [8, 9]:

• For minor health problems (rash, respiratory problems, sinus infections, swelling and bruising), the patient should go to a primary care provider;
• For cases with low acuity (accidents and falls; bleeding/cuts – without much bleeding, but requiring sutures; difficulty in breathing (mild to moderate asthma, diagnosis services, including X-rays and laboratory tests, eye irritation and redness, fever or flu, minor bone and fractures, moderate back problems, severe sore throat or cough, rash and infection, sprains and strains, urinary tract infections, vomiting, diarrhea or dehydration) treatment should also be sought in primary care offices.

The purpose of integrating marketing within the Emergency Reception Unit (ERU) is to meet both the objectives of the medical organization in the role of a provider and the patient as a consumer. Healthcare marketers have the responsibility to identify the objectives of the participants in the process and ease their accessibility, including the improvement of the image of the health organization, along with promoting a coherent perception of the quality of the health care, the professionalism of the staff, certain subjective patient values.

Both evolving consumer requirements and regulatory requirements and technological challenges, which have evolved into major and rapid market changes, are forcing medical organizations to completely rethink their organizational structure and culture, implementing the principles of efficient marketing management practices, consumer-based oriented, providing them with quality experiences in line with their expectations [10].

## Conclusion

Today’s context triggered the creation of a high capacity for innovation, flexibility, and stability in the health system, especially in the active effort to implement or finalize the information implementation processes, in real-time, by implementing healthcare marketing management strategies.

Educating patients is the key to the proper management of an ER.

As marketing is the philosophy that deals with the satisfaction of needs and wants of the healthcare consumers, its integration into the ER may bring benefits both for the medical organization, the patients, and, implicitly, for the specialized staff. As the results have shown, although 40.5% of the ER physicians suffer from burnout, the healthcare organization supports them in all their activities, making 25.7% of the ER physicians satisfied and joyful.

## Acknowledgments

### Conflict of interest

The authors declare that there is no conflict of interest.
